# Supplementary material for: Network control energy reductions under DMT relate to serotonin receptors, signal diversity, and subjective experience
Source: Commun Biol. 2025 Apr 18;8:631. doi: 10.1038/s42003-025-08078-9 (PMC12008288; doi:10.1038/s42003-025-08078-9)
Supplement: Supplementary file 1 — Supplemental Information [file 42003_2025_8078_MOESM1_ESM.pdf]

Supplemental Information:
Network control energy reductions under DMT relate to serotonin receptors, signal diversity, and subjective experience

S. Parker Singleton[1*], Christopher Timmermann[2], Andrea I. Luppi[3], Emma Eckernäs[4], Leor Roseman[2], Robin L. Carhart-Harris[2,5], Amy Kuceyeski[1,6]

[1] Department of Computational Biology, Cornell University, 304E Atkinson Hall, Ithaca, USA, 14853

[2] Center for Psychedelic Research, Department of Brain Science, Imperial College London, Du Cane Road, London, United Kingdom, W12 0NN

[3] Montreal Neurological Institute, 3801 Rue University, Montreal, Canada, H3A 2B4

[4] Unit for Pharmacokinetics and Drug Metabolism, Department of Pharmacology, Sahlgrenska Academy at University of Gothenburg, Box 431, Gothenburg, Sweden, 405 30

[5] Psychedelics Division, Neuroscape, University of California San Francisco, 675 Nelson Rising Lane, San Francisco, USA, 94158

[6] Department of Radiology, Weill Cornell Medicine, 1300 York Ave, New York, USA, 10065

*Corresponding author
Email address: sps253@cornell.edu (S. Parker Singleton)

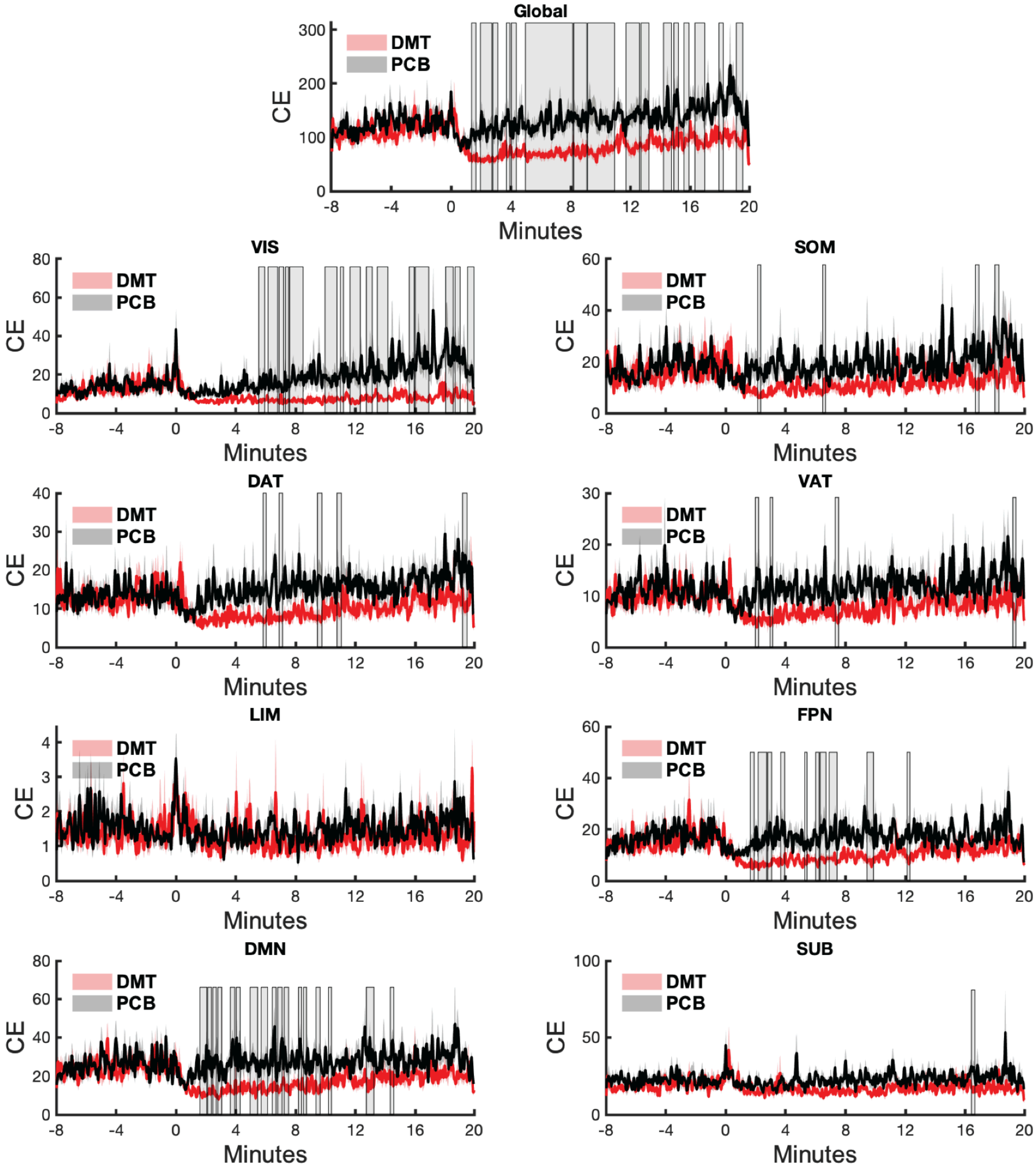

SI Fig. 1: Subnetwork level comparisons in post-injection control energy. Here, we replicate the main text results from Figure 2a. At the top, we reprint the global results from Figure 2a for the reader's convenience. Below, we repeat the analysis for each of the well-characterized intrinsic connectivity subnetworks of Yeo and colleagues[1], plus an additional subcortical one. The most prominent reductions in control energy occur in the visual, frontoparietal, and default mode (sub)networks. Each subnetwork's control energy was obtained by summing control energy over all nodes assigned to that network. Gray boxes reflect

cluster-corrected significant time-points (n = 14 subjects; see *Control energy calculation* and *Network-level control energy analyses* for details). VIS = visual; SOM = somatomotor; DAT = dorsal attention; VAT = ventral attention; LIM = limbic; FPN = frontoparietal; DMN = default mode; SUB; subcortex.

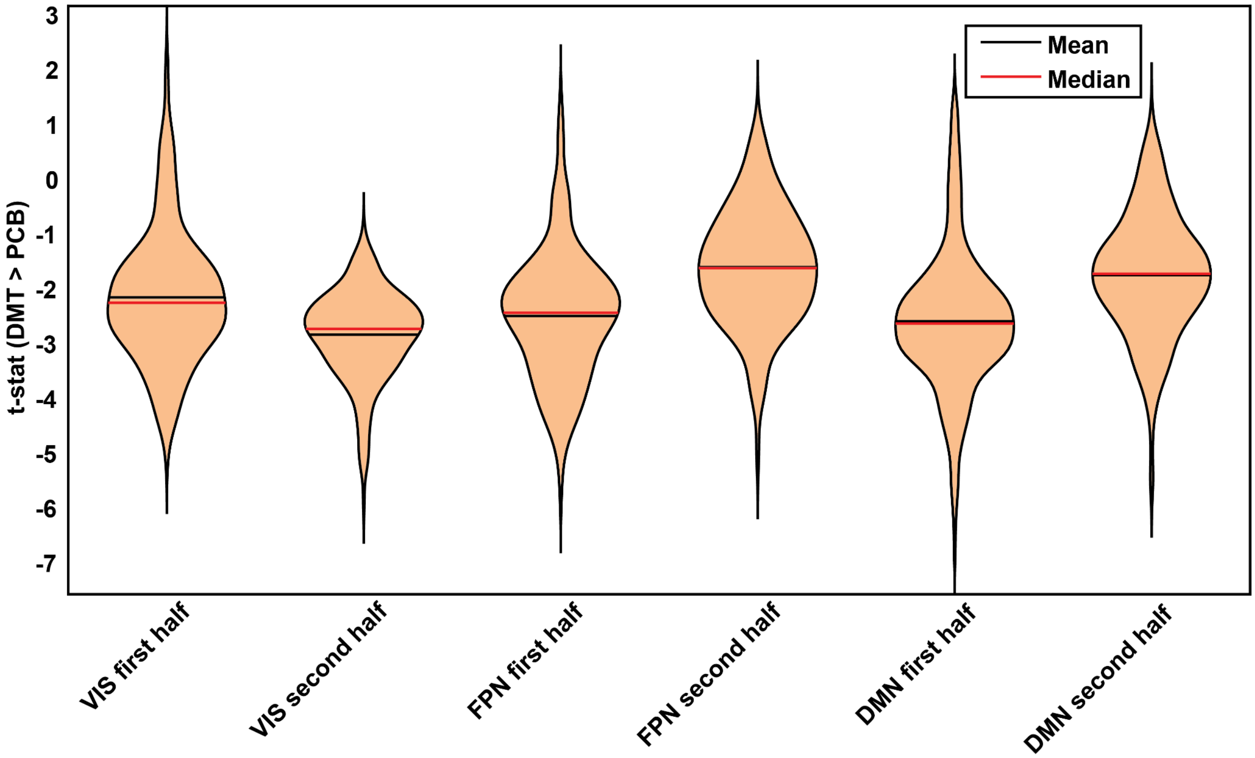

SI Fig. 2: Subnetwork control energy differences in the first and second half of post-injection scanning. For each subnetwork that was significantly impacted by DMT (SI Figure 1), we plot the average t-statistic for transitions occurring in the first ten minutes after injection (first half) and the last ten minutes after injection (second half). We note that the frontoparietal (FPN) and default mode networks (DMN) are most greatly impacted in the first ten minutes after injection, whereas the opposite is true for the visual network (VIS).

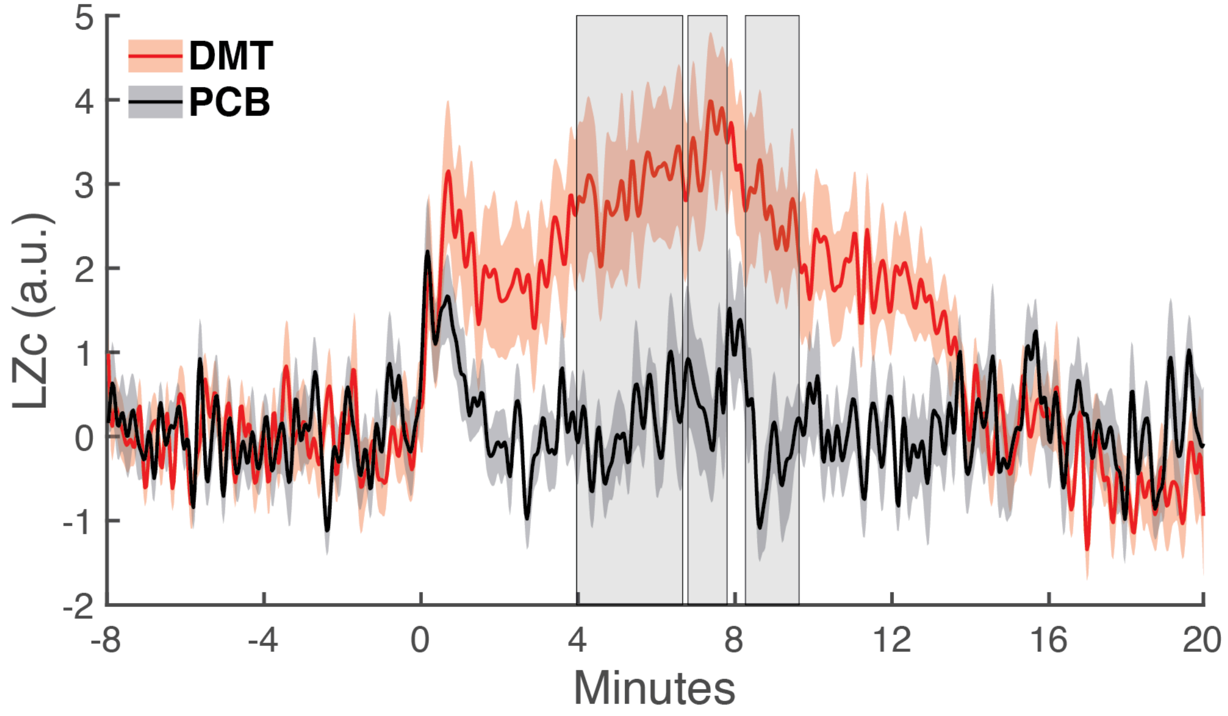

SI Fig. 3: LZ_c comparison. A direct comparison of LZ_c from EEG under DMT and placebo. Gray boxes reflect cluster-corrected significant time-points (n = 14 subjects: see *EEG preprocessing and signal diversity calculation* for details). Solid lines are group means and corresponding shaded boundaries reflect the standard error of the mean (SEM). Grey boxes reflect cluster-corrected significant time-points. LZ_c = Lempel-Ziv complexity; a.u. = arbitrary units.

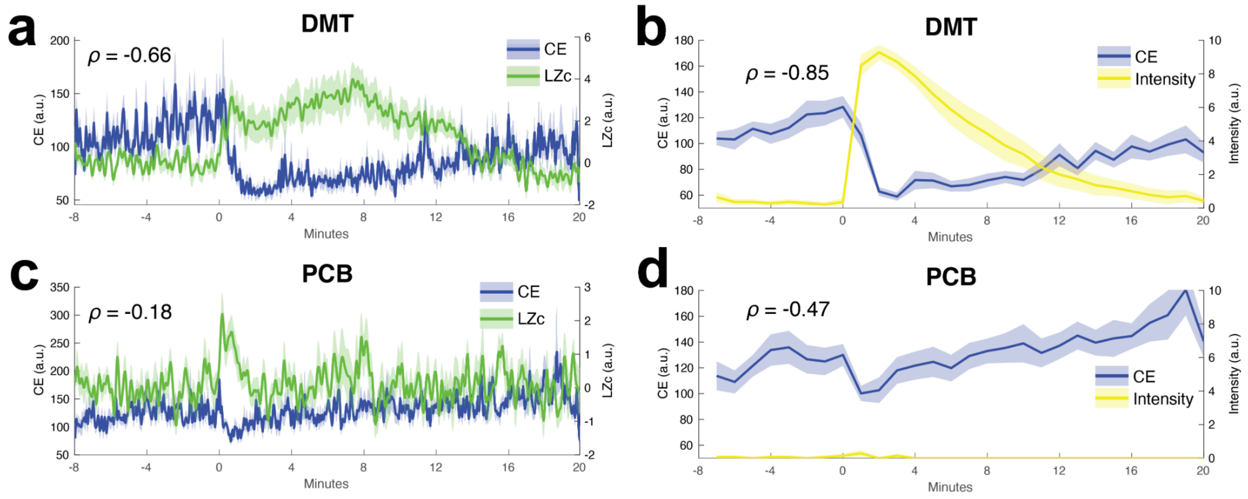

SI Fig. 4: Condition-specific correlations. Correlations from Fig. 2b and 2c, in each condition separately. (a) Group-level correlation between global CE and LZ_c for the DMT condition (n = 838 time-points; $p_{perm} < 0.0001$). (b) Group-level correlation between global CE and intensity for

the DMT condition (n = 28 time-points; $p_{perm} < 0.0001$). (c) Group-level correlation between global CE and LZ_c for the placebo condition (n = 838 time-points; $p_{perm} < 0.0001$). (d) Group-level correlation between global CE and intensity for the placebo condition (n = 28 time-points; $p_{perm} = 0.0097$). Solid lines are group means and corresponding shaded boundaries reflect the standard error of the mean (SEM). CE = control energy; LZ_c = Lempel-Ziv complexity; a.u. = arbitrary units.

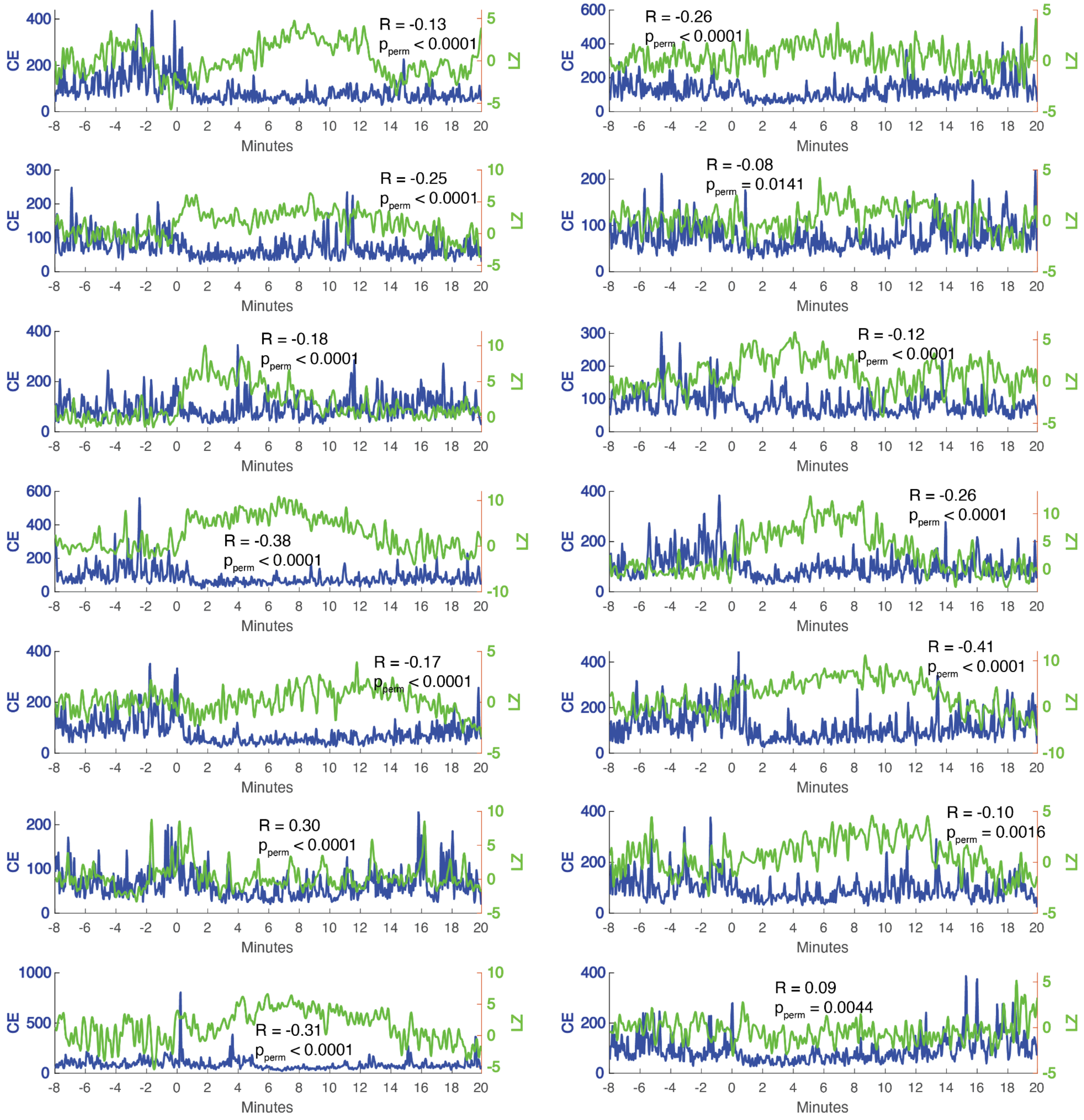

SI Fig. 5: Individual-level correlations between global CE and LZ_c for the DMT condition. n = 838 time-points; CE = control energy; LZ_c = Lempel-Ziv complexity.

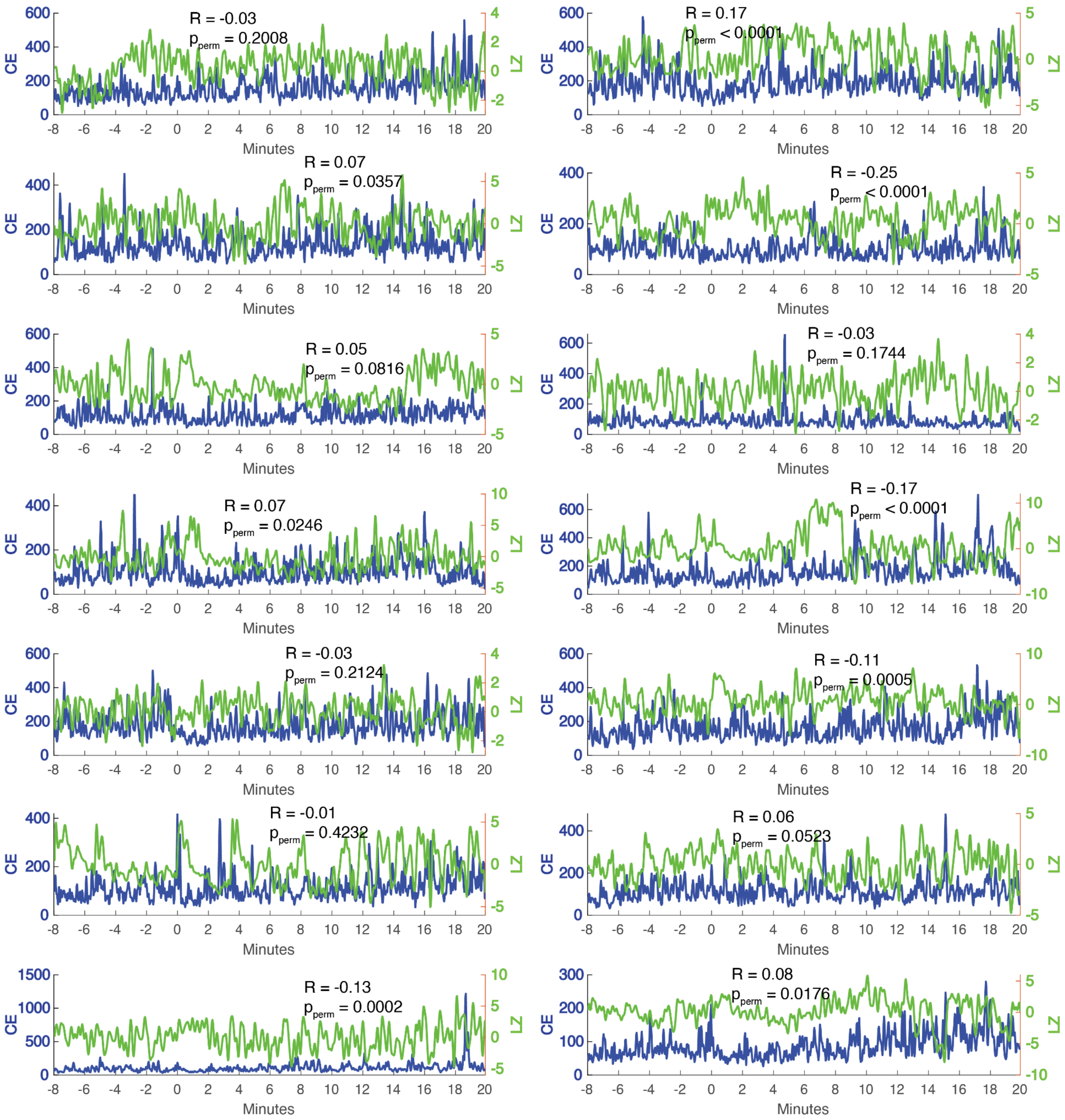

SI Fig. 6: Individual-level correlations between global CE and LZ_c for the placebo condition. n = 838 time-points; CE = control energy; LZ_c = Lempel-Ziv complexity.

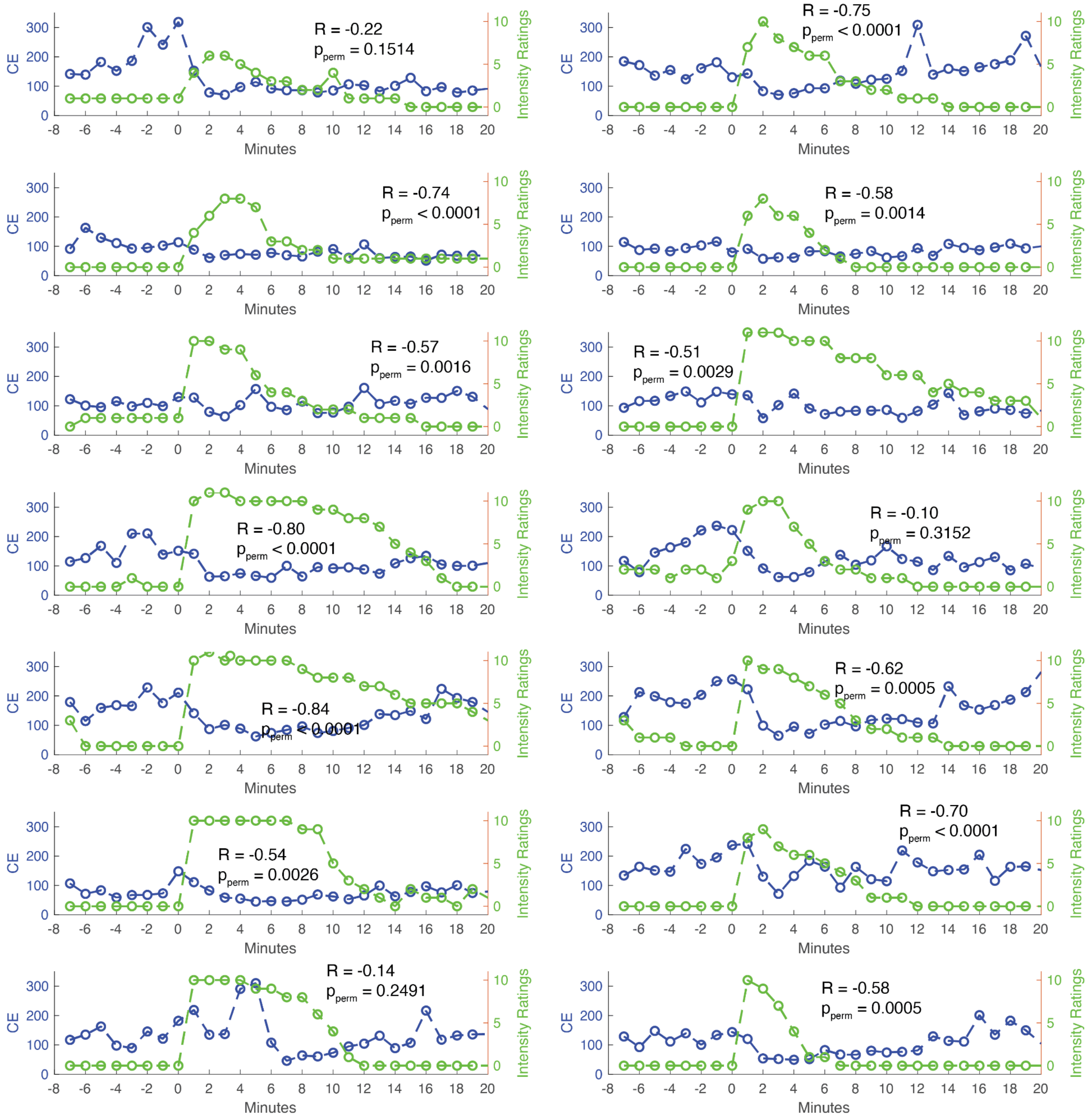

SI Fig. 7: Individual-level correlations between global CE and subjective drug intensity for the DMT condition. n = 28 time-points; CE = control energy.

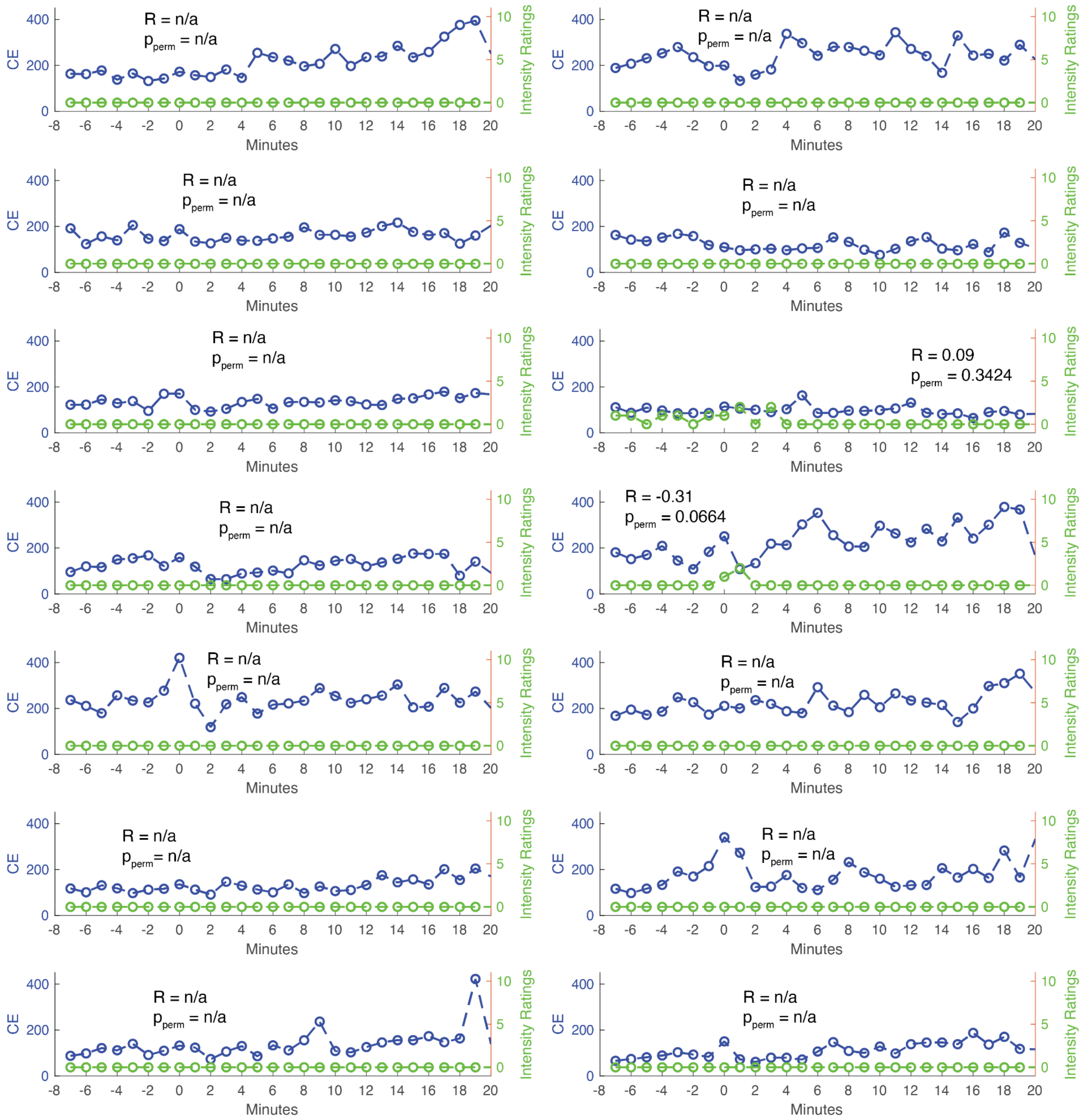

SI Fig. 8: Individual-level correlations between global CE and subjective drug intensity for the placebo condition. Several individuals rated intensity at 0 for every time-point and correlations could not be computed for these subjects. n = 28 time-points; CE = control energy.

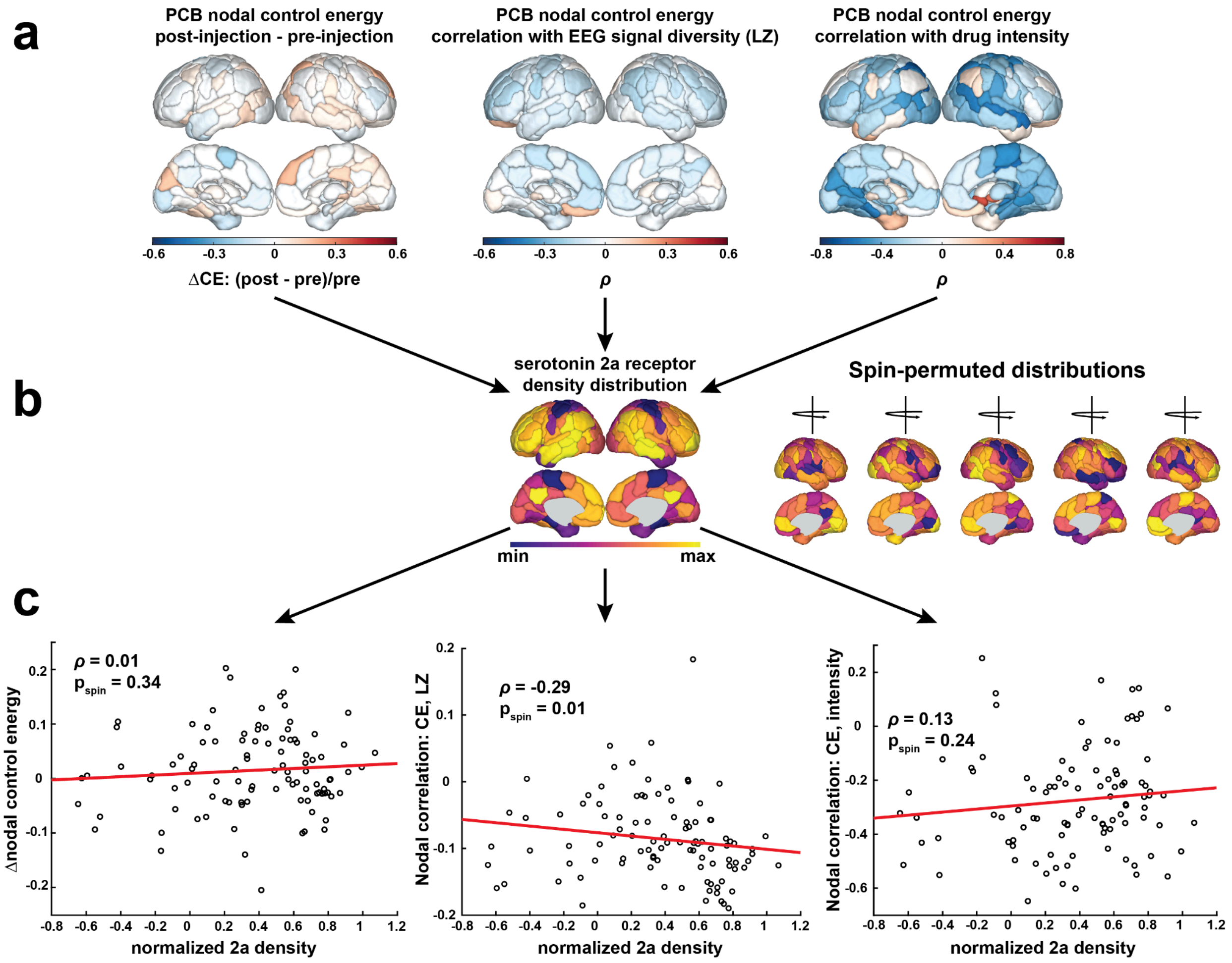

SI Fig. 9: Replication of analyses from Main Text Fig. 3 using the placebo scans instead of DMT scans. (a) Regional control energy metrics. (left) The change in regional control energy in the 8 minutes after PCB injection, relative to the 8 minutes prior to the injection. (middle) Each region's control energy time-series over the course of the full 28 minute PCB scans correlated with global signal diversity from EEG during the same scans. (right) Regional control energy during the PCB scans was averaged over one minute windows corresponding to the timing of subjective drug intensity ratings from separate scans. The windowed control energy time-series for each region was then correlated with the subjective drug intensity ratings. **(b)** Each of the regional metrics in (a) were then correlated with the cortical spatial map of the serotonin 2a receptor derived from PET. The strength of these correlations were compared against null correlations with 10,000 cortical spin permutations of the 2a receptor map. **(c)** Scatter plots of the three cortical regions' metrics (n = 100 regions) from (a) and serotonin 2a receptor density from (b).

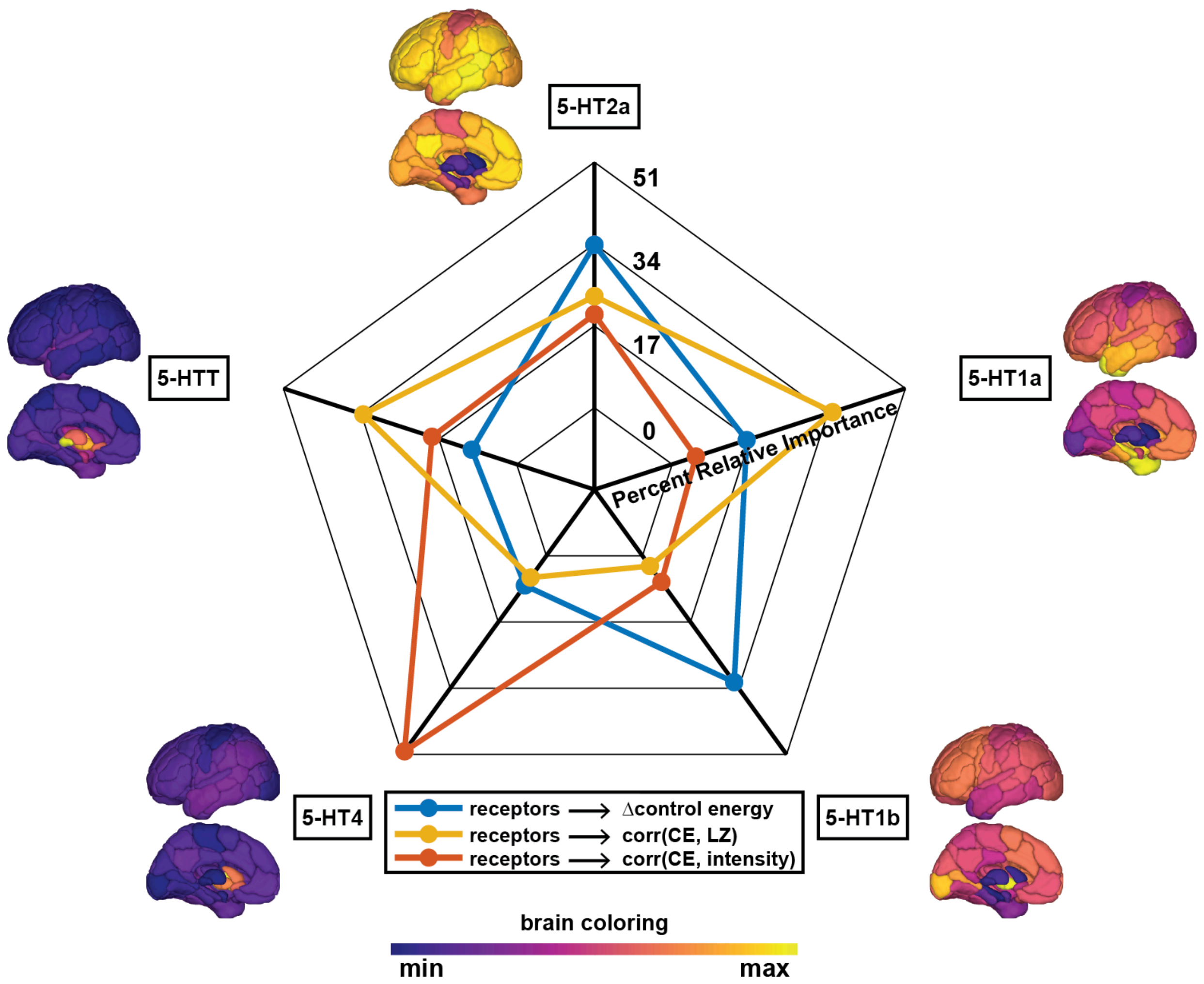

SI Fig. 10: Replication of analyses from Main Text Fig. 4 using the placebo scans instead of DMT scans. Three separate dominance analyses were performed using cortical values from five PET-derived serotonin receptor and transporter spatial densities[2] as input variables and each cortical metric from Fig. 3a as the output. Dominance analysis assesses the relative importance of each input in explaining the output variable's variance while controlling for the contributions of other predictors in multiple regressions. Displayed is the percent relative importance given to each receptor/transporter map for explaining the variance in each cortical metric, as determined by dominance analysis. n = 116 regions; 5-HT = serotonin (5-hydroxytryptamine).

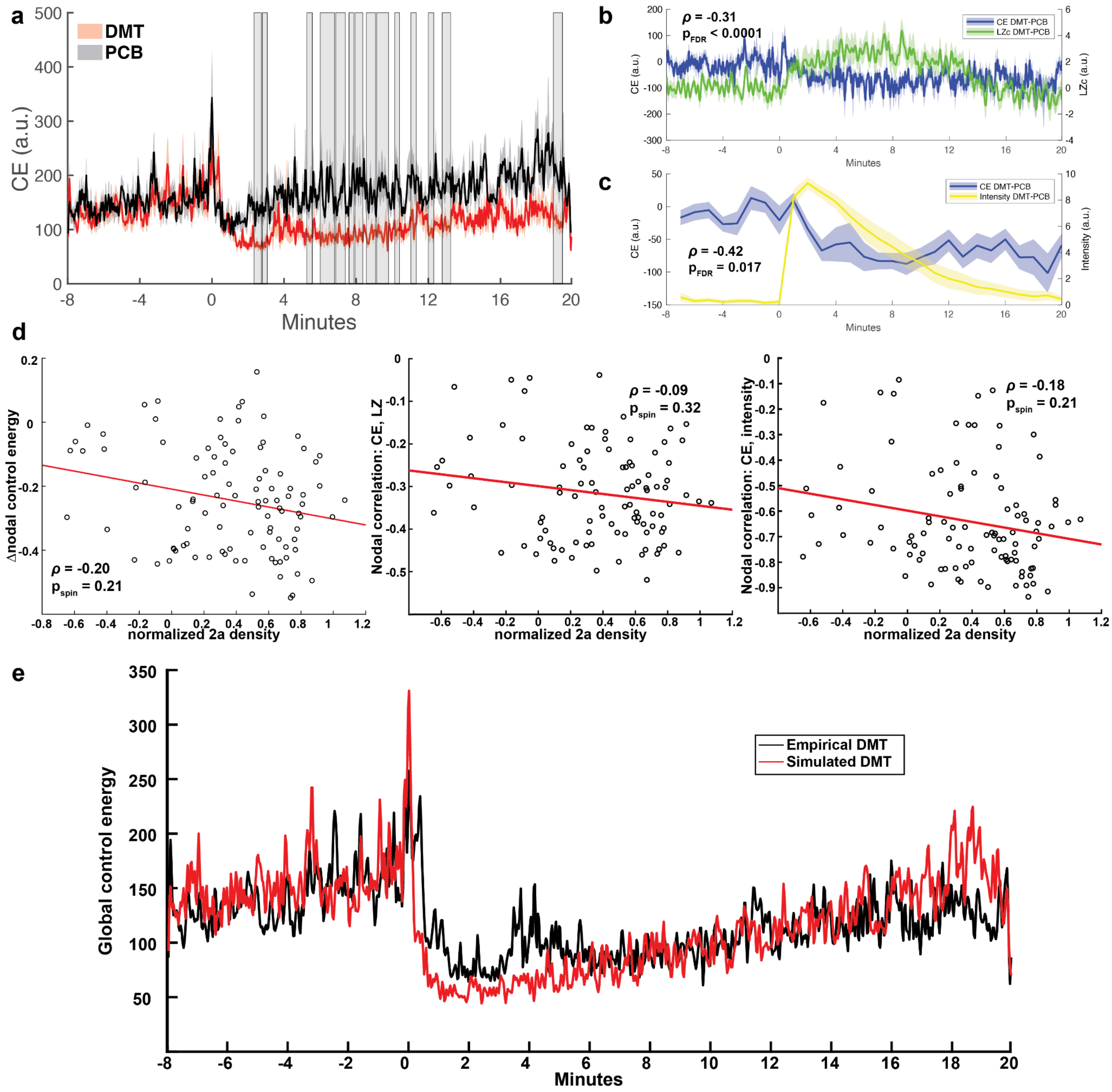

SI Fig. 11: Replication of main results without the use of global signal regression. (a-c) Main results from Figure 2. (d) Main results from Figure 3c. (e) Main results from Figure 5. Solid lines are group means and corresponding shaded boundaries reflect the standard error of the mean (SEM). Grey boxes reflect cluster-corrected significant time-points. CE = control energy; LZ_c = Lempel-Ziv complexity; a.u. = arbitrary units.

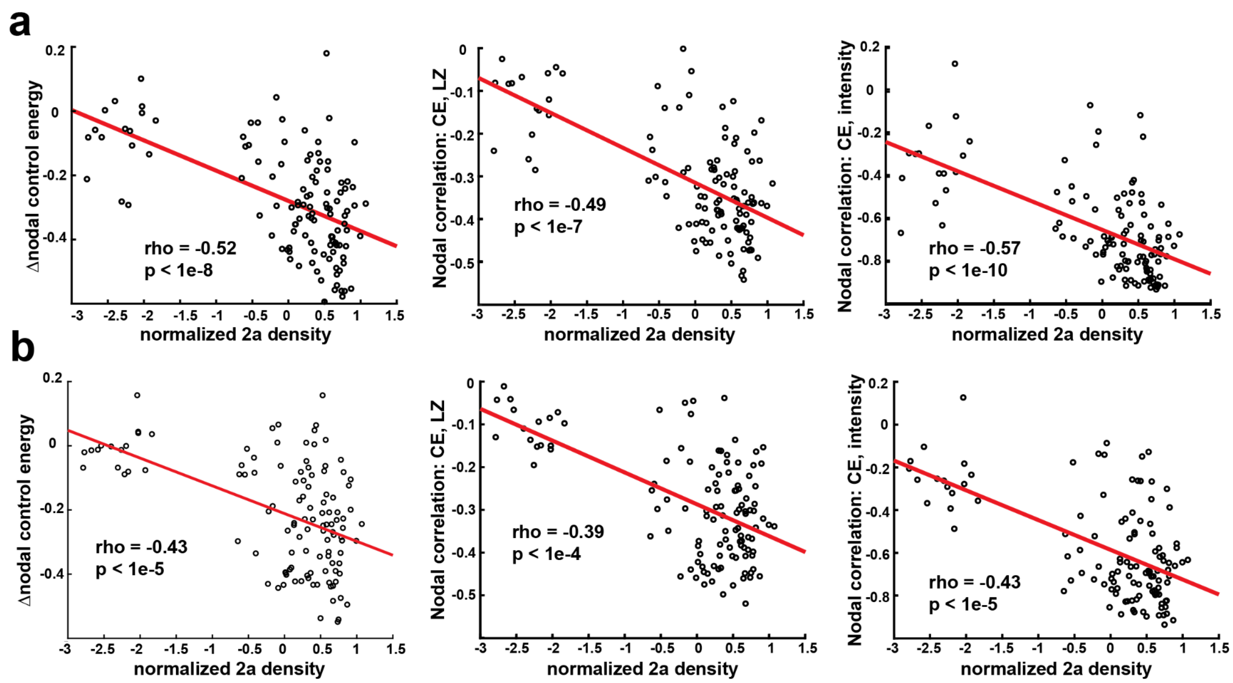

SI Fig. 12: Nodal metric correlations including the subcortex. (a) Scatter plots from Main Figure 3c (with global signal regression), repeated with the subcortical regions included. (b) Scatter plots from SI Figure 2d (no global signal regression), repeated with the subcortical regions included. Spearman rank correlations, uncorrected p-values (n = 116 regions). CE = control energy; LZ_c = Lempel-Ziv complexity.

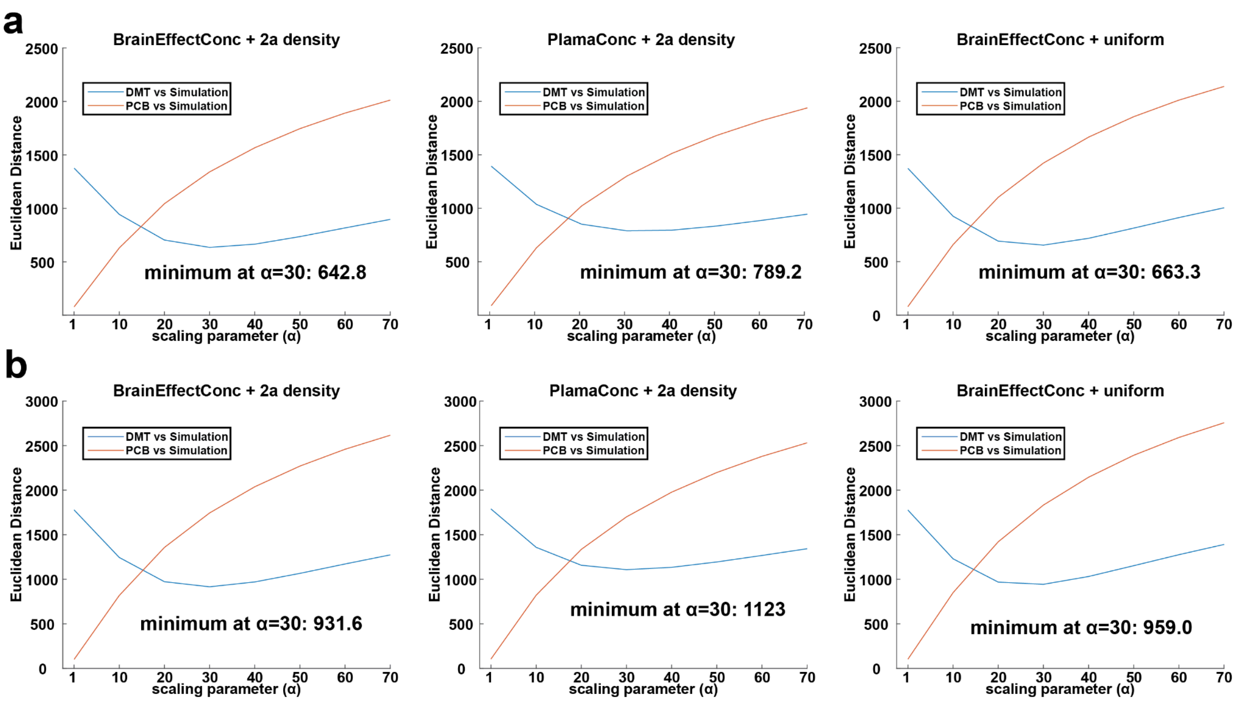

SI Figure 13: Optimization of scaling parameter for model simulations. (a) Main text simulations (with global signal regression). (b) Simulations without the use of global signal regression. (left) Optimization of scaling parameter for the main model presented in the main text, which uses simulated brain effect compartment concentrations for DMT's impact over time, and the serotonin 2a density for spatial differences. (middle) The first comparison model which uses simulated plasma concentration rather than brain effect concentration. (right) The second comparison model, which removes the effect of the serotonin 2a spatial map in the original model by adding uniform control in its place. In this second model, the same amount of overall control is given to the system as in the original simulation. We note the Euclidean distance quantifying model error is minimal in the first model's simulations for both sets of data.

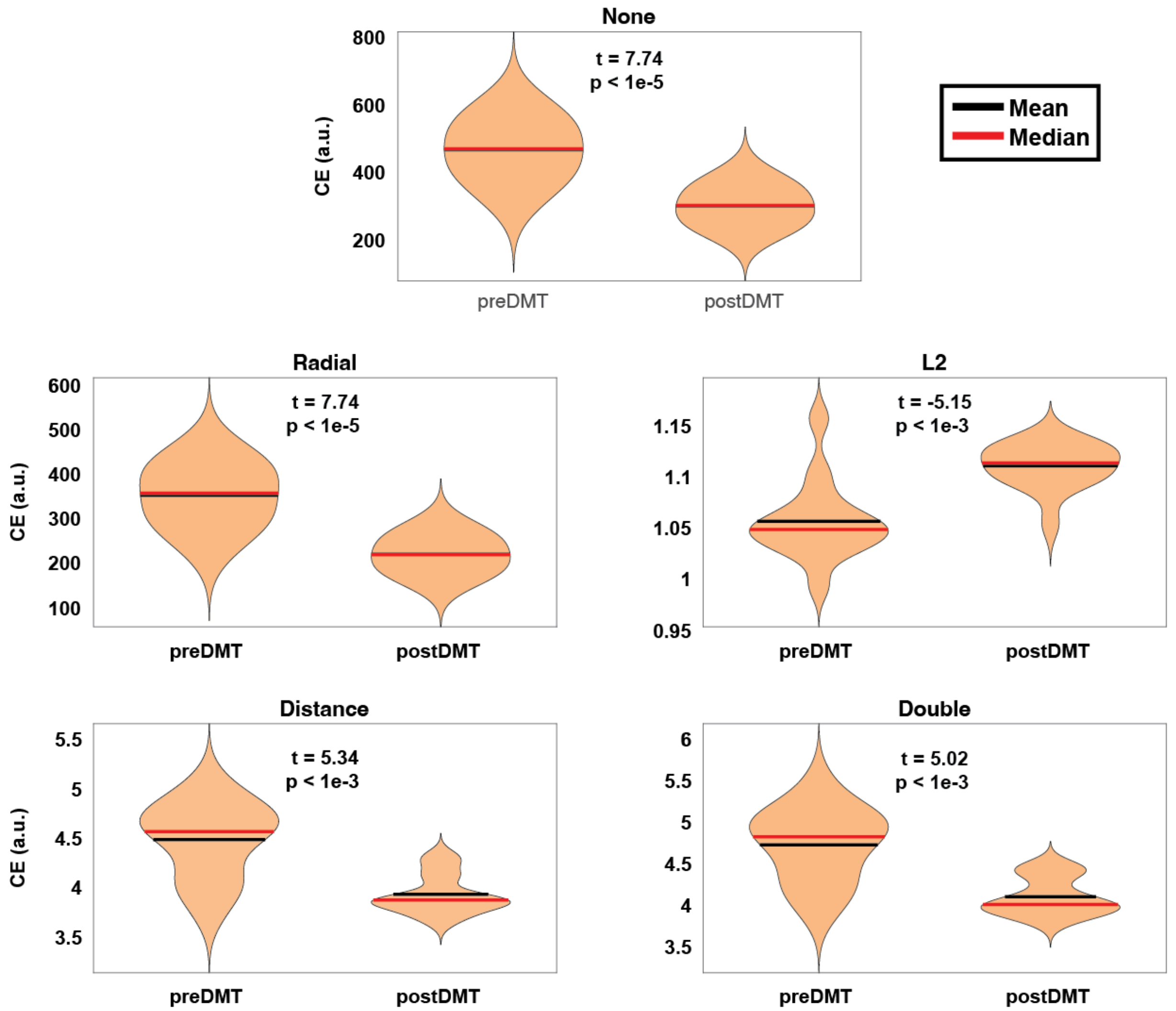

SI Figure 14: Comparison of average global control energy over the 8 minutes prior to injection to the 8 minutes following injection, using different methods of brain activity normalization. All methods of activity normalization result in a significant decrease in control energy after DMT injection, except for L2 normalization, which results in a significant increase in control energy. This suggests that when accounting for the magnitude of activity, states are more difficult to reach through a network control process. This is likely related to the concurrent psychedelic effect of decreased signal magnitude and increased temporal entropy. When magnitude is normalized and its effects removed from the energy calculations, the decreased autocorrelation between adjacent BOLD activity dominates the energy calculations and results in increased control energy. When both magnitude and inter-state distance are accounted for (double normalization), DMT still decreases control energy. This suggests that when both factors are accounted for, states are still closer together through a network diffusion process after DMT compared to before DMT. None = no additional state normalization applied beyond fMRI preprocessing (main text version). Radial = final states are normalized so that they are unit distance from initial states. L2 = all states are normalized by their L2 magnitudes. Distance = each pair of initial and final states are normalized by the magnitude of their inter-state distance.

Double = first L2 normalization is applied to all states, then distance normalization is applied to all pairs of initial and final states. n = 14 subjects; CE = control energy; a.u. = arbitrary units.

References:

1. Yeo, B. T. *et al.* The organization of the human cerebral cortex estimated by intrinsic functional connectivity. *J. Neurophysiol.* **106**, 1125–1165 (2011).
2. Beliveau, V. *et al.* A High-Resolution *In Vivo* Atlas of the Human Brain's Serotonin System. *J. Neurosci.* **37**, 120–128 (2017).
