# Supplementary material for: Network control energy reductions under DMT relate to serotonin receptors, signal diversity, and subjective experience
Source: Commun Biol. 2025 Apr 18;8:631. doi: 10.1038/s42003-025-08078-9 (PMC12008288; doi:10.1038/s42003-025-08078-9)
Supplement: Supplementary file 4 — Reporting Summary [file 42003_2025_8078_MOESM4_ESM.pdf]

nature portfolio

Corresponding author(s): S. Parker Singleton

Last updated by author(s): Apr 8, 2025

Reporting Summary

Nature Portfolio wishes to improve the reproducibility of the work that we publish. This form provides structure for consistency and transparency in reporting. For further information on Nature Portfolio policies, see our Editorial Policies and the Editorial Policy Checklist.

Statistics

For all statistical analyses, confirm that the following items are present in the figure legend, table legend, main text, or Methods section.

n/a	Confirmed	
☐	☒	The exact sample size (*n*) for each experimental group/condition, given as a discrete number and unit of measurement
☐	☒	A statement on whether measurements were taken from distinct samples or whether the same sample was measured repeatedly
☐	☒	The statistical test(s) used AND whether they are one- or two-sided<br>*Only common tests should be described solely by name; describe more complex techniques in the Methods section.*
☐	☒	A description of all covariates tested
☐	☒	A description of any assumptions or corrections, such as tests of normality and adjustment for multiple comparisons
☐	☒	A full description of the statistical parameters including central tendency (e.g. means) or other basic estimates (e.g. regression coefficient) AND variation (e.g. standard deviation) or associated estimates of uncertainty (e.g. confidence intervals)
☐	☒	For null hypothesis testing, the test statistic (e.g. *F*, *t*, *r*) with confidence intervals, effect sizes, degrees of freedom and *P* value noted<br>*Give P values as exact values whenever suitable.*
☒	☐	For Bayesian analysis, information on the choice of priors and Markov chain Monte Carlo settings
☒	☐	For hierarchical and complex designs, identification of the appropriate level for tests and full reporting of outcomes
☐	☒	Estimates of effect sizes (e.g. Cohen's *d*, Pearson's *r*), indicating how they were calculated

Our web collection on statistics for biologists contains articles on many of the points above.

Software and code

Policy information about availability of computer code

Data collection	No new data were used in this analysis. The original data are available at: https://github.com/timmer500/DMT_Imaging
Data analysis	All code required to reproduce this analysis are available at: https://github.com/singlesp/DMT_NCT

For manuscripts utilizing custom algorithms or software that are central to the research but not yet described in published literature, software must be made available to editors and reviewers. We strongly encourage code deposition in a community repository (e.g. GitHub). See the Nature Portfolio guidelines for submitting code & software for further information.

Data

Policy information about availability of data

All manuscripts must include a data availability statement. This statement should provide the following information, where applicable:

- Accession codes, unique identifiers, or web links for publicly available datasets
- A description of any restrictions on data availability
- For clinical datasets or third party data, please ensure that the statement adheres to our policy

Data used in this analysis were published alongside the original study (Timmermann et al. 2023). Code to reproduce this analysis is available at: https://github.com/singlesp/DMT_NCT.

Research involving human participants, their data, or biological material

Policy information about studies with human participants or human data. See also policy information about sex, gender (identity/presentation), and sexual orientation and race, ethnicity and racism.

Reporting on sex and gender	This study was not powered to report on sex or gender differences.
Reporting on race, ethnicity, or other socially relevant groupings	This study was not powered to report on race, ethnicity, or other socially relevant groupings.
Population characteristics	In total, 20 healthy human participants completed all study visits (7 female, mean age = 33.5 years, SD = 7.9)
Recruitment	Participants were recruited via word of mouth.
Ethics oversight	This study study was approved by the National Research Ethics (NRES) Committee London – Brent and the Health Research Authority and was conducted under the guidelines of the revised Declaration of Helsinki (2000), the International Committee on Harmonisation Good Clinical Practices guidelines, and the National Health Service Research Governance Framework.

Note that full information on the approval of the study protocol must also be provided in the manuscript.

Field-specific reporting

Please select the one below that is the best fit for your research. If you are not sure, read the appropriate sections before making your selection.

☒ Life sciences ☐ Behavioural & social sciences ☐ Ecological, evolutionary & environmental sciences

For a reference copy of the document with all sections, see nature.com/documents/nr-reporting-summary-flat.pdf

Life sciences study design

All studies must disclose on these points even when the disclosure is negative.

Sample size	A sample size of 20 individuals was chosen based on prior work in the field.
Data exclusions	Six out of 20 participants were discarded from group analyses due to excessive head movement during the 28 minute DMT scans (>20% of scrubbed volumes with a scrubbing threshold of frame-wise displacement (FD) of 0.4 (Power et al. 2014)), leaving 14 for analysis.
Replication	Our findings were consistent with and without the use of global signal regression on the fMRI data and when using in-scanner motion co-variates.
Randomization	Individuals were randomized to two conditions: those receiving placebo first followed by DMT on the first visit (and then the opposite on the second visit) and those receiving DMT first followed by placebo on the first visit (and then the opposite on the second visit)
Blinding	Participants were blind to the order of administration but study personnel were not. This allowed for study personnel to respond quickly to any psychological distress that may have arisen during DMR administration.

Reporting for specific materials, systems and methods

We require information from authors about some types of materials, experimental systems and methods used in many studies. Here, indicate whether each material, system or method listed is relevant to your study. If you are not sure if a list item applies to your research, read the appropriate section before selecting a response.

Materials & experimental systems

n/a	Involved in the study	
☒	☐	Antibodies
☒	☐	Eukaryotic cell lines
☒	☐	Palaeontology and archaeology
☒	☐	Animals and other organisms
☒	☐	Clinical data
☒	☐	Dual use research of concern
☒	☐	Plants

Methods

n/a	Involved in the study	
☒	☐	ChIP-seq
☒	☐	Flow cytometry
☐	☒	MRI-based neuroimaging

Plants

Seed stocks

Report on the source of all seed stocks or other plant material used. If applicable, state the seed stock centre and catalogue number. If plant specimens were collected from the field, describe the collection location, date and sampling procedures.

Novel plant genotypes

Describe the methods by which all novel plant genotypes were produced. This includes those generated by transgenic approaches, gene editing, chemical/radiation-based mutagenesis and hybridization. For transgenic lines, describe the transformation method, the number of independent lines analyzed and the generation upon which experiments were performed. For gene-edited lines, describe the editor used, the endogenous sequence targeted for editing, the targeting guide RNA sequence (if applicable) and how the editor was applied.

Authentication

Describe any authentication procedures for each seed stock used or novel genotype generated. Describe any experiments used to assess the effect of a mutation and, where applicable, how potential secondary effects (e.g. second site T-DNA insertions, mosiacism, off-target gene editing) were examined.

Magnetic resonance imaging

Experimental design

Design type

resting-state

Design specifications

Volunteers participated in two testing days, separated by 2 weeks. On each testing day, participants arrived and were tested for drugs of abuse and were involved in 2 separate scanning sessions. In this initial session (task-free) they received intravenous (IV) administration of either placebo (10 mL of saline) or 20 mg DMT (in fumarate form dissolved in 10 mL of saline)-injected over 30s, and then flushed with 10 mL of saline over 15s-in a counterbalanced order (half of the participants received placebo in the first scanning session and DMT in the second scanning session, and the other half received DMT in the first scanning session and placebo in the second scanning session). The first session always consisted of continuous resting-state scans which lasted 28 minutes with DMT/placebo administered at the end of the 8th minute. Participants laid in the scanner with their eyes closed (an eye mask was used to prevent eyes from opening), while EEG activity was recorded. The second scanning session then followed the first after participants had fully returned to baseline (at least two hours later) with the same procedure as the initial session, except on this occasion participants were asked to rate the subjective intensity of drug effects every minute in real time. Specifically, the ratings were collected by playing an audio cue (“Intensity from 0 to 10”) through MR-compatible earphones which was followed by the participants’ verbal response collected via an MR-compatible microphone. On the second testing day, participants who received placebo for the first scanning session on the first testing day then received DMT for the first scanning session and vice versa. Due to the differential tolerance of biological and subjective effects to DMT in humans (R. J. Strassman, Qualls, and Berg 1996), carryover effects from one testing day to the other are not expected, and the subjective intensity experienced for the same dose is expected to be roughly similar between testing days.

This article only analyzes the resting-state scans in which no intensity ratings were asked, but uses the intensity ratings for correlational analyses.

Images were acquired in a 3T MRI (Siemens Magnetom Verio syngo MR B17) using a 12-channel head coil for compatibility with EEG acquisition. Functional imaging was performed using a T2*-weighted BOLD sensitive gradient echo planar imaging sequence with the following parameters: repetition time (TR) = 2000ms, echo time (TE) = 30ms, acquisition time (TA) = 28.06 mins, flip angle (FA) = 80°, voxel size = 3.0 x 3.0 x 3.0mm3, 35 slices, interslice distance = 0mm. Whole-brain T1-weighted structural images were also acquired.

EEG was recorded inside the MRI during image acquisition at 31 scalp sites following the 10-20 convention with an MR compatible BrainAmp MR amplifier (BrainProducts, Munich, Germany) and an MR-compatible cap (BrainCap MR; BrainProducts GmbH, Munich, Germany). This system referenced all electrodes to FCz and AFz served as the ground electrode. Two additional ECG channels were used to improve heart rate acquisition for artifact minimization during EEG preprocessing, and all impedances were kept below 20kΩ. EEG was sampled at 5 kHz and with a hardware 250 Hz low-pass filter. EEG-MR clock synchronization was ensured using the Brain Products SyncBox hardware. Additional recordings of 5 min eyes-closed resting-state were performed outside the scanner before DMT/placebo was administered in order to determine the profile of EEG activity in the time and frequency domain and ensure that artifact minimization procedures achieved a similar profile.

Behavioral performance measures

The ratings were collected by playing an audio cue (“Intensity from 0 to 10”) through MR-compatible earphones which was followed by the participants’ verbal response collected via an MR-compatible microphone

Acquisition

Imaging type(s)

functional

Field strength

3T

Sequence & imaging parameters

Images were acquired in a 3T MRI (Siemens Magnetom Verio syngo MR B17) using a 12-channel head coil for compatibility with EEG acquisition. Functional imaging was performed using a T2*-weighted BOLD sensitive gradient echo planar imaging sequence with the following parameters: repetition time (TR) = 2000ms, echo time (TE) = 30ms,

	acquisition time (TA) = 28.06 mins, flip angle (FA) = 80°, voxel size = 3.0 x 3.0 x 3.0mm3, 35 slices, interslice distance = 0mm.
Area of acquisition	whole brain

Diffusion MRI ☐ Used ☒ Not used

Preprocessing

Preprocessing software	AFN (Cox 1996)I, FSL (Smith et al. 2004), ANTS (Avants, Tustison, and Song 2009), Freesurfer (Dale, Fischl, and Sereno 1999)
Normalization	non-linear registration to 2mm MNI brain
Normalization template	2mm MNI
Noise and artifact removal	Preprocessing steps consisted of 1) de-spiking (3dDespike, AFNI (Cox 1996)), 2) slice time correction (3dTshift, AFNI), 3) motion correction (3dvolreg, AFNI) by registering each volume to the most similar volume, in the least squares sense, to all others (minimizing the amount of transformation required for each volume), 4) brain extraction (BET, FSL (Smith et al. 2004)), 5) rigid body registration to anatomical scans, 6) non-linear registration to 2mm MNI brain (Symmetric Normalization (SyN), ANTS (Avants, Tustison, and Song 2009)), 7) scrubbing - using an FD threshold of 0.4 - with scrubbed volumes being replaced with the mean of the surrounding volumes. Additional preprocessing steps included: 8) spatial smoothing (FWHM) of 6mm (3dBlurInMask, AFNI), 9) band-pass filtering between 0.01 to 0.08 Hz (3dFourier, AFNI), 10) linear and quadratic de-trending (3dDetrend, AFNI), 11) regressing out 9 nuisance regressors (all nuisance regressors were bandpass filtered with the same filter as in step 9), out of these, 6 were motion-related (3 translations, 3 rotations) and 3 were anatomically-related. Specifically, the anatomical nuisance regressors were: a) ventricles (Freesurfer (Dale, Fischl, and Sereno 1999), eroded in 2 mm space), b) draining veins (FSL's CSF minus Freesurfer's ventricles, eroded in 1mm space (Smith et al. 2004; Dale, Fischl, and Sereno 1999)), and c) local white matter (WM) (FSL's WM minus Freesurfer's subcortical gray matter structures, eroded in 2mm space (Smith et al. 2004; Dale, Fischl, and Sereno 1999). Lastly, global signal regression was performed.
Volume censoring	volumes with a frame-wise displacement greater than 0.4 were replaced with the mean of surrounding volumes

Statistical modeling & inference

Model type and settings	Cluster-based permutation testing was used to compare control energy between placebo and DMT at each time point with a significance threshold of 0.05 (two-tailed) (Oostenveld 2011). All correlational analyses were performed using Spearman-rank correlations. P-values were generated against permutation null models.
Effect(s) tested	DMT's impacts on control energy, and its correlation with signal-diversity and subjective intensity over time. Regional effects were correlated with the serotonin 2a receptor distribution.

Specify type of analysis: ☐ Whole brain ☐ ROI-based ☒ Both

Anatomical location(s): Augmented 232 region Schaefer-Tian atlas

Statistic type for inference (See Eklund et al. 2016)	n/a
Correction	P-values were corrected using Benjamini-Hochberg.

Models & analysis

n/a	Involved in the study
☒	☐ Functional and/or effective connectivity
☒	☐ Graph analysis
☒	☐ Multivariate modeling or predictive analysis
